# Optical hydrogen sensing with high-Q guided-mode resonance of Al_2_O_3_/WO_3_/Pd nanostructure

**DOI:** 10.1038/s41598-023-28204-z

**Published:** 2023-01-17

**Authors:** Daria P. Kulikova, Yevgeniy M. Sgibnev, Georgiy M. Yankovskii, Eugeny D. Chubchev, Evgeniy S. Lotkov, Daria A. Ezenkova, Alina A. Dobronosova, Aleksandr S. Baburin, Ilya A. Rodionov, Igor A. Nechepurenko, Alexander V. Baryshev, Alexander V. Dorofeenko

**Affiliations:** 1 Dukhov Research Institute of Automatics (VNIIA), Moscow, Russia; 2 Faculty of Physics, Lomonosov Moscow State University, Moscow, Russia; 3 FMN Laboratory, Bauman Moscow State Technical University, Moscow, Russia; 4 Kotelnikov Institute of Radioengineering and Electronics RAS, Moscow, Russia; 5 Moscow Institute of Physics and Technology, Dolgoprudny, Moscow, Russia; 6 Institute for Theoretical and Applied Electromagnetics RAS, Moscow, Russia

**Keywords:** Sensors and biosensors, Optical sensors

## Abstract

Nanostructure based on a dielectric grating (Al_2_O_3_), gasochromic oxide (WO_3_) and catalyst (Pd) is proposed as a hydrogen sensor working at the room temperature. In the fabricated structure, the Pd catalyst film was as thin as 1 nm that allowed a significant decrease in the optical absorption. A high-Q guided-mode resonance was observed in a transmission spectrum at normal incidence and was utilized for hydrogen detection. The spectra were measured at 0–0.12% of hydrogen in a synthetic air (≈ 80% ${\text{N}}_{2}$ and 20% ${\text{O}}_{2}$). The detection limit below 100 ppm of hydrogen was demonstrated. Hydrogen was detected in the presence of oxygen, which provides the sensor recovery but suppresses the sensor response. Sensor response was treated by the principal component analysis (PCA), which effectively performs noise averaging. Influence of temperature and humidity was measured and processed by PCA, and elimination of the humidity and temperature effects was performed. Square root dependence of the sensor response on the hydrogen concentration (Sievert’s law) was observed. Sensor calibration curve was built, and the sensor resolution of 40 ppm was found. Long term stability of the sensor was investigated. Particularly, it was shown that the sensor retains its functionality after 6 months and dozens of acts of response to gas.

## Introduction

Green energy demand inspires the development of hydrogen technologies, which allow for efficient, sustainable and zero-emission energy management^1^. This also creates a need for hydrogen monitoring with the hydrogen detectors and analyzers being the key elements. While the most common are resistive gas sensors based on thin films of metal oxides, they have working temperatures from 100 to 400 °C and limited selectivity^2–5^. To avoid the necessity of heating, optical sensors based on gasochromic properties of metal oxides (WO_3_, NiO, etc.) adjacent to a catalyst (Pd, Pt)^6–8^ are elaborated. These sensors use the property of materials to change the extinction coefficient and/or refractive index as a result of reaction with a gas^9^. Optical gas sensors of H_2_ and other gases demonstrate a detection limit of tens and hundreds of ppm^10^, which is enough for most applications.

Tungsten trioxide has a pronounced response to hydrogen that makes it one of the most promising platforms for optical H_2_ detection^11–14^. One of the main challenges for developing efficient hydrogen sensors based on metal oxides is substantial decrease in sensor response in oxygen-containing atmosphere. This is caused by the reverse reaction with oxygen, which influences performance of both optical and resistive hydrogen sensors^15–17^. To overcome this problem, a higher sensitivity is necessary. Various approaches, including doping and nanostructuring, were used to enhance gasochromic response of WO_3_-based sensors to hydrogen^7,18,19^. One of the main goals of nanostructuring is the increase of the optical response while keeping the building layers thin to provide a rapid response. Also, the optical path along the surface of the nanostructure should be large enough to make the integral response strong enough. For this reason, the structure supporting the guided-mode resonance seems to be well suitable. Guided-mode resonance is widely used for the development of various sensors thanks to tunability of a resonant wavelength and high sensitivity^20–25^.

Essential part of any sensor is an algorithm for processing the sensor data. In many cases, the sensitivity may be enhanced by a proper processing procedure^26,27^. In the case of optical sensors, the data processing is of particular importance because of a complex spectral response of this class of sensors^28,29^.

In the present work, an optical guided-mode resonance hydrogen sensor based on a 1D Al_2_O_3_/WO_3_/Pd nanostructure working in an oxygen-containing atmosphere was proposed and studied. The catalytic Pd film was as thin as 1 nm to avoid significant optical losses. We experimentally demonstrated an existence of absorption resonances in the nanostructure and showed that the ultrathin catalytic film was enough for an effective function of the sensor: a high sensitivity and a fast response. The sensor resolution of 40 ppm was demonstrated with the sensor data processing by the principal component analysis (PCA). Selectivity to hydrogen was proved by PCA when changing humidity of the exposing atmosphere and heating the sensor. It was found that the sensor retained its function after several months of intensive use, although the response became somewhat weaker and slower.

## Sample preparation and characterization

The functionality of the proposed nanostructure was provided by a gasochromic ${\text{WO}}_{3}$ film with Pd catalyst. This choice was governed by a strong response of ${\text{WO}}_{3}$ to hydrogen and by its low optical absorption in the absence of hydrogen, which makes possible an achievement of a high-Q optical response and a high sensitivity. The absorption inherent to Pd was minimized by the use of an ultrathin (1 nm) coating.

**Figure 1:**
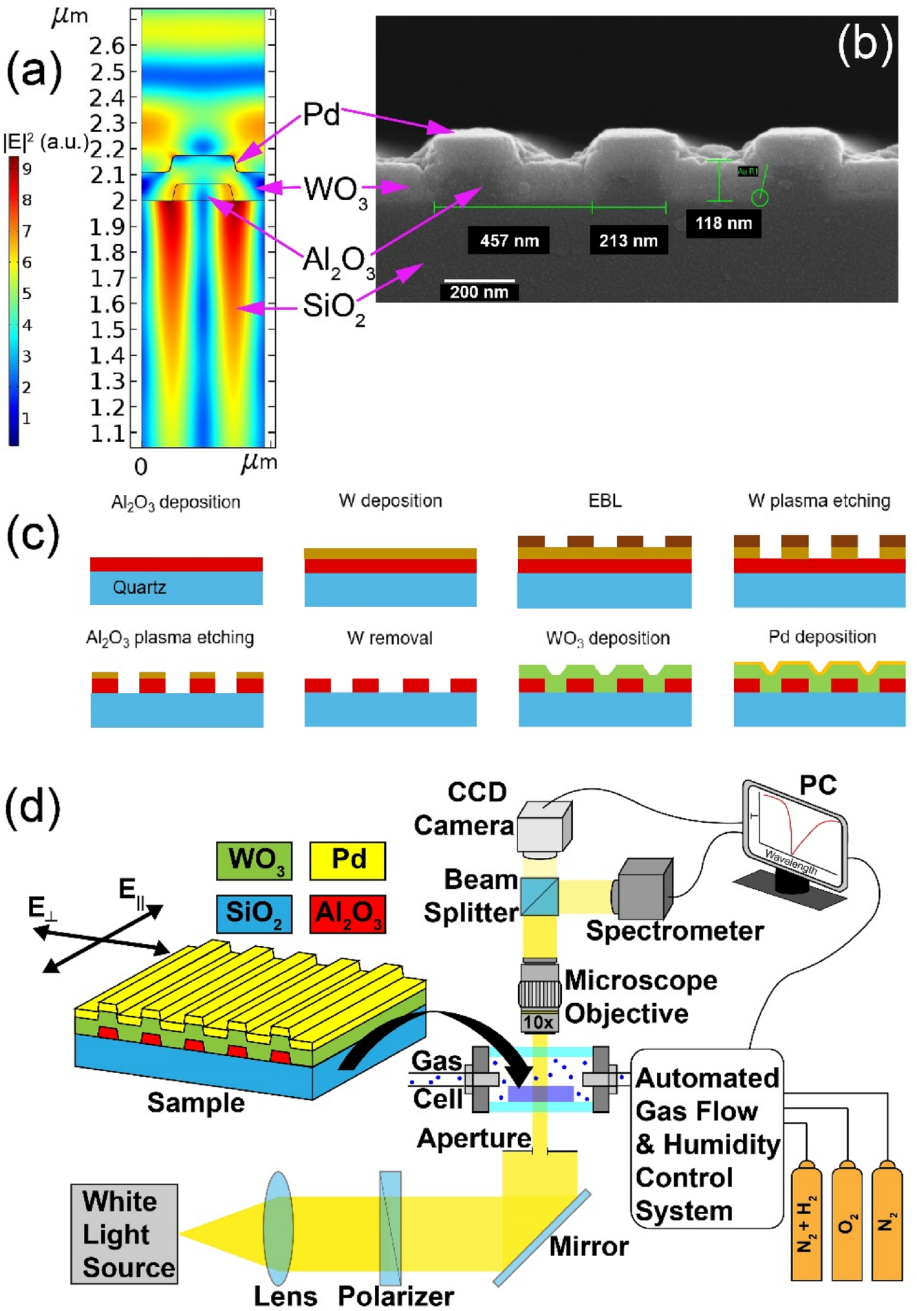
Electric field distribution in the 1D structure at a resonant frequency for E_⊥_ polarization (**a**), SEM image of the fabricated 1D structure (**b**), dielectric structure fabrication process^36^ (**c**), scheme of experimental setup for measurement of transmission spectra (**d**).

A nanostructure comprising an Al_2_O_3_ grating, ${\text{WO}}_{3}$ film, and ultrathin Pd coating on a quartz substrate (Fig. 1a) was optimized with respect to the geometric parameters. The optimization procedure maximized a sensor sensitivity $\left| {\delta T/\left( {\delta n + i\,\delta k} \right)} \right|$, where $\delta T$—a change in the transmittance at a resonance wavelength resulting from a change $\delta n + i\,\delta k$ in the complex refractive index of WO_3_, (i.e., the simultaneous change in the real (*n*) and imaginary (*k*) parts). Electromagnetic simulations were carried out in COMSOL Multiphysics. The result of optimization strongly depended on the ratio of $\delta n$ and $\delta k$, because $\delta n$ influences mostly spectral shift of the resonance, whereas $\delta k$ defines its magnitude. One can find that under exposure to hydrogen a change in *k* of WO_3_ at $\lambda$ near 700 nm is twice as one in *n*^30–34^, $\delta k\sim 2\,\delta n$. The permittivities of the constituent materials were found from preliminary experiments (ellipsometry and transmittance spectra of nonstructured layers and bilayers samples, see Ref.^35^ for details). The optimized structure was a 1D grating of 85 × 220-nm (height × width) sized Al_2_O_3_ ridges deposited with a 460 nm pitch on a UV-grade quartz substrate. The grating was then covered by 110-nm thick WO_3_ and 1-nm thick Pd layers. For such design, a standing wave is formed in the 1D structure at the resonance wavelength (Fig. 1a).

A 10 kW e-beam evaporator (Angstrom Engineering, Canada) with a base pressure lower than 3 × 10^−8^ Torr was applied to fabricate all the thin films^37,38^. First, 85 nm thick alumina films (Fig. 1c) were deposited on 25 mm × 25 mm quartz substrates at a rate of 0.25 nm/s. The thicknesses of alumina films were retrieved from an ellipsometer measurements (Sentech Instruments GmbH, Germany).

For the patterning of the alumina film, a 150 nm tungsten (W) film hard masks were deposited at a rate of 0.10 nm/s. Next, thin film of e-beam resist polymethyl methacrylate (PMMA) A4 (MicroChem, Westborough, MA, USA) was spin-coated at a rate of 1500 rpm for 60 s, and baked at 150 °C for 1 min. Its thickness was near 300 nm as estimated from measurements by a reflectometer. The 1D grating topologies were fabricated by 50 keV electron beam lithography (EBL). The EBL exposure dose was 850 µC/cm^2^. The samples were developed in the methyl isobutyl ketone (MIBK) for 60 s. Following reactive ion etching process consisted of two steps. First, the tungsten hard masks were selectively etched using mixture of fluorine gases. After the first step, the e-beam resist masks were removed. On the second step, Al_2_O_3_ layers etching was carried out using chlorine gases (BCl_3_ and Cl_2_ mixture) plasma^39^ with the following tungsten mask removal in a fluorine gases plasma. After that, the Al_2_O_3_ gratings were covered by an e-beam evaporation with 110 nm thick tungsten oxide (WO_3_) layer at a rate of 0.50 nm/s and oxygen partial pressure of $5 \times 10^{ - 5}$ Torr and 1 nm palladium (Pd) layer at a rate of 0.025 nm/s. The thicknesses of tungsten oxide films were retrieved from the ellipsometer measurements of a control sample. The thicknesses of palladium films were controlled by quartz crystal monitoring system*.* The fabricated nanostructure (hereafter, sample) is shown in Fig. 1b, and its elemental analysis is given in Supplementary (Fig. S2).

Transmission spectra of the sample were measured at normal incidence using the setup shown in Fig. 1d. Collimated polarized beam from a white light source (XWS-65, TRDC, Russia) passed through an aperture and the sample placed in a gas cell with quartz windows, heater and thermocouple to control both chemical composition of the atmosphere and temperature. Transmitted light was collected with a 10X objective (NA = 0.28) and analyzed by a thermo-electric cooled fiber optic spectrometer (AvaSpec-ULS, Avantes). Visualization of the sample orientation was realized with a CCD camera. Nitrogen, oxygen and hydrogen gas mixture was prepared with mass flow controllers (Bronkhorst). Humidity was controlled with an evaporation mixer together with mass flow controllers, and pressure controllers were combined in an automated gas flow and humidity control system. A commercial humidity sensor was used for monitoring of humidity. Measurement of transmission spectra of the sensor was performed under a constant gas mixture flow of 100 ml/min at atmospheric pressure.

**Figure 2:**
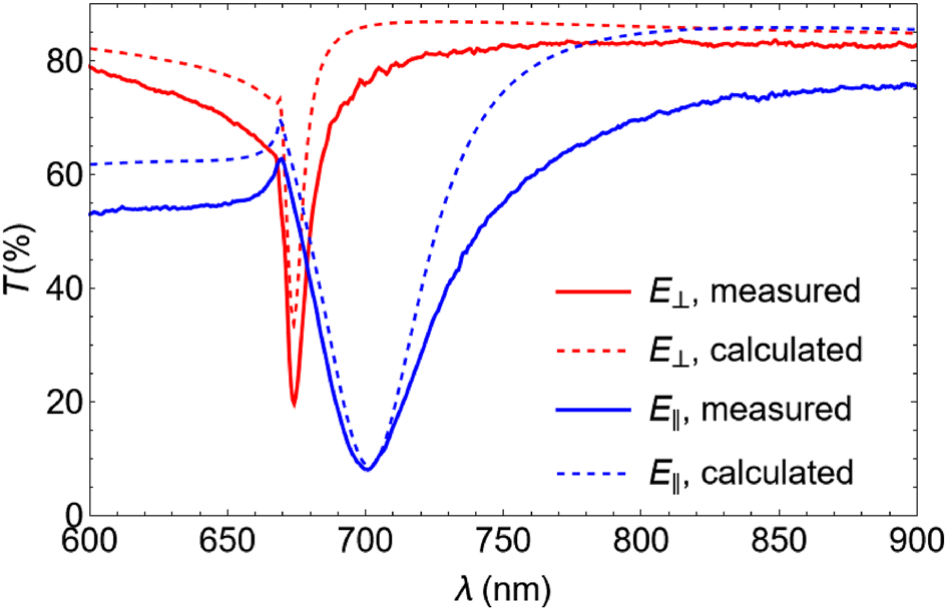
Measured (solid) and calculated (dashed) transmission spectra of the sensing element for the E_⊥_ (red) and E_||_ (blue) polarizations.

To perform the sample characterization, transmission spectra of the sensing element were measured in both polarizations, parallel (E_||_) and perpendicular (E_⊥_) to the Al_2_O_3_ stripes. The results revealed the presence of a resonance dip (guided-mode resonance^40,41^), in accordance with the preliminary calculations, which were in a good agreement with the calculated spectra (Fig. 2). The resonance dip in the E_⊥_-polarized transmission spectrum was characterized by a smaller FWHM if compared to that in the E_||_-polarized spectrum. This was due to the lower scattering and, consequently, lower radiative losses of the E_⊥_-polarized guided mode compared to the E_||_ one. The transmission dip is associated with the first-order diffraction condition of 1D grating, *λ* = *n*_*eff*_* D*, where *n*_*eff*_ is the effective index of the guided mode and *D* is the grating period*.* One can see that the difference between the experimental and calculated spectra was larger for the E_||_ polarization, which was a consequence of a larger effect of fabrication imperfections causing parasitic light scattering.

To interprete the transmission spectra of the sensor and determine the sensor response, we employed the principal component analysis, see Supplementary for more details. In our case, the first principal component $PC_{1} \left( \lambda \right)$ was enough to describe the observed spectral change in the transmission spectrum $\Delta T\left( \lambda \right)$ caused by interaction with hydrogen: $\Delta T\left( \lambda \right) \approx a_{PC1} PC_{1} \left( \lambda \right)$, the amplitude of the principal component $a_{PC1}$ being considered as the sensor response. The sensor response defined in such manner demonstrates a good stability against spectral noise compared to analysis of $\Delta T$ at a single wavelength because PCA uses a spectral range involving the resonance, $\lambda_{1} \le \lambda \le \lambda_{2}$. We have chosen $\lambda_{1} = 667.9$ nm and $\lambda_{2} = 682.7$ nm by minimizing a standard error (see “Concentration dependence of the sensor response“, particularly, Fig. 4d). On the other hand, the value of $a_{PC1}$ has a clear interpretation—it is proportional to an average change in the transmission spectra due to the presence of hydrogen, $\sqrt {\left\langle {\Delta T^{2} } \right\rangle } = \left[ {\int\limits_{{\lambda_{1} }}^{{\lambda_{2} }} {\left( {T\left( {{\text{air}} + {\text{H}}_{2} } \right) - T\left( {{\text{air}}} \right)} \right)^{2} d\lambda /\left( {\lambda_{2} - \lambda_{1} } \right)} } \right]^{1/2}$ [see Eq. (S5) in Supplementary]. For this reason, the sensor response is hereafter given both in terms of $a_{PC1}$ and, more clearly, in terms of $\sqrt {\left\langle {\Delta T^{2} } \right\rangle }$.

## Sensor response in oxygen-free and oxygen-containing atmospheres

The sensor response to hydrogen results from the hydrogen dissociation by the Pd catalyst, ${\text{H}}_{2} \to 2{\text{H}}^{ + } + 2e^{ - }$, and subsequent reduction of the tungsten ions, ${\text{W}}^{6 + } \mathop\rightarrow ^{{{\text{H}}^{ + } }} {\text{W}}^{5 + }$^7^. The sensor recovery is carried out by oxygen, ${\text{W}}^{5 + } \mathop\rightarrow ^{{{\text{O}}_{2} }} {\text{W}}^{6 + }$, which oxidizes the tungsten ions reduced at the previous step. One of the main challenges in the development of efficient metal oxide hydrogen sensors is substantial decrease in the sensor response in oxygen-containing atmosphere^16^ since the oxygen concentration (near 20%) is much higher than that of hydrogen (not exceeding 0.12% in our experiments). As a result, the oxidation reaction shifts the equilibrium closely to the initial state.

**Figure 3:**
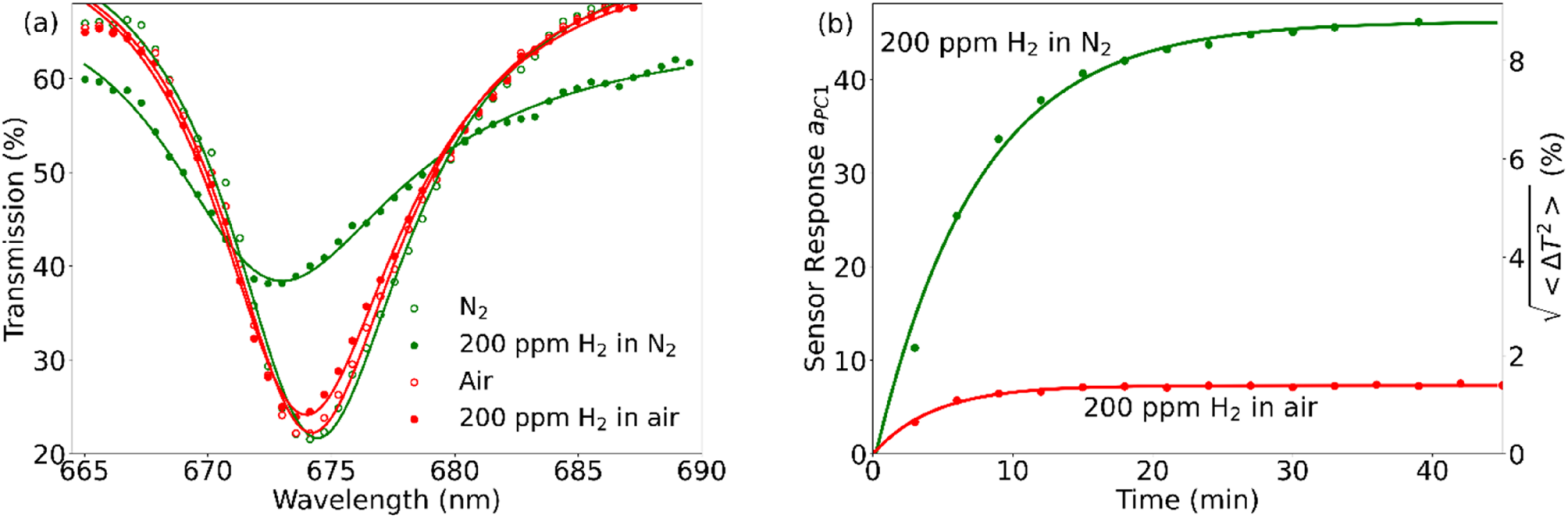
Sensor response to 200 ppm hydrogen in nitrogen or in air: spectral response (**a**) and time-dependent response (**b**) shown in terms of the amplitude of the first principal component, $a_{PC1}$, and of a corresponding average spectral change in transmittance, $\sqrt {\left\langle {\Delta T^{2} } \right\rangle }$ (right scale). Curves show the exponential fit of the experimental data. The spectra are measured in the E_⊥_ polarization.

In our experiments, the sensor performance was studied both in nitrogen and in a synthetic air (≈ 80% N_2_ and 20% O_2_, hereafter, air). These cases differ principally because the recovery process is absent in the oxygen-free atmosphere, where the sensor saturates and gives a maximal response, whereas in the presence of oxygen, a dynamic equilibrium between the reduction–oxidation reactions is observed. The presence of hydrogen resulted in a complex spectral response (Fig. 3a), which consisted in an increase in transmission at the resonance wavelength and in a blueshift of the resonance. These are due to changes in the real and imaginary parts of the refractive index of WO_3_ produced by the gasochromic coloration process. Each spectrum was processed to find the amplitude of the first principal component, $a_{PC1}$ (see “Sample preparation and characterization” and Supplementary). As a result, a dynamic sensor response, $a_{PC1}$ vs time, was obtained (Fig. 3b). Sensor response to 200 ppm of hydrogen was 6–7 times lower in air than in nitrogen. The experimental dependence was fitted with an exponential function, $a_{PC1} = a_{0} - a_{1} \exp \left( { - t/\tau } \right)$. The sensor response time $\tau$ was found to decrease from $7.5 \pm 0.4$ min in nitrogen to $4.3 \pm 0.3$ min in air. Corresponding values of the response amplitude $a_{1}$ were $48.4 \pm 1.2$ and $7.23 \pm 0.2$ ($9.28 \pm 0.23$% and $1.39 \pm 0.04$% in terms of $\sqrt {\left\langle {\Delta T^{2} } \right\rangle }$, respectively). Thus, in the presence of oxygen, somewhat faster but considerably weaker response was observed.

## Concentration dependence of the sensor response

**Figure 4:**
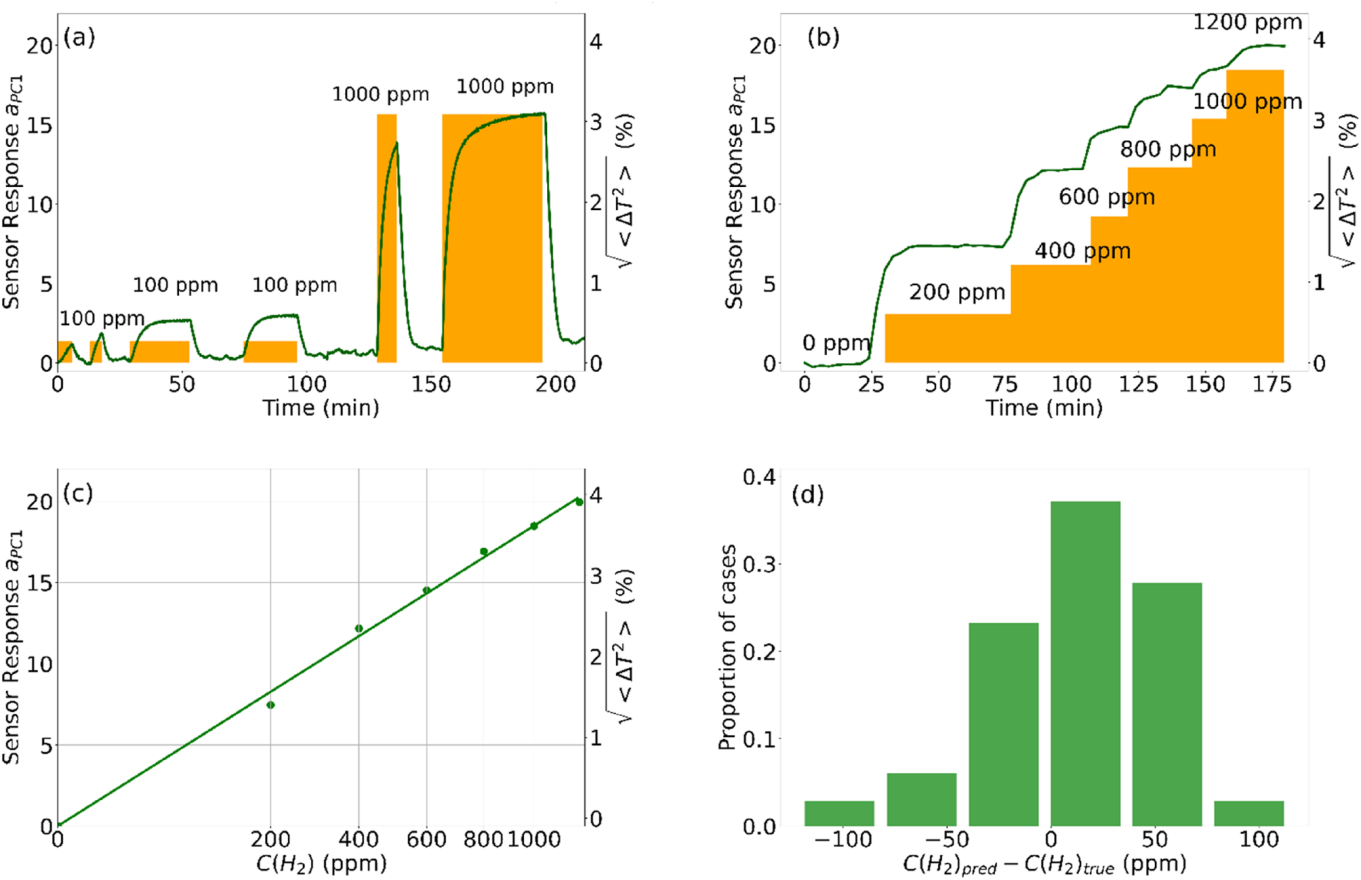
Response of the sensor to 100 ppm and 1000 ppm of hydrogen switched on and off (**a**) and to a “ladder” of hydrogen concentrations increased from 200 to 1200 ppm in air (**b**). (**c**) Dependence of the sensor response on the hydrogen concentration in air; shown versus the square root scale for hydrogen concentrations. (**d**) Resulting error hystogram.

For practical use, where a target gas concentration must be defined, it is necessary to measure a calibration dependence. To get this dependence, we carried out two series of measurements. In the first one, reduction was done at two hydrogen concentrations (100 ppm and 1000 ppm) in air during time intervals lasting from several minutes to some dozens of minutes, each interval following by oxidation in air (Fig. 4a). These data were used for generation of the first principal component; a wavelength dependence of $PC_{1} \left( \lambda \right)$ is shown in Supplementary (Fig. S3). In the second series, the sensor was exposed to a ladder of successively growing concentrations (Fig. 4b), each step was kept for ≈ 2 min. The measured spectra were processed with the use of the principal component $PC_{1} \left( \lambda \right)$ obtained from the first series. As a result, the calibration dependence was determined (Fig. 4c).

We observed such feature of hydrogen sensors as a growth of sensitivity (i.e., of a derivative of $a_{PC1}$ with respect to the hydrogen concentration) at low hydrogen concentrations. Particularly, one can see in Fig. 4a,b that the response to lower (100 and 200 ppm) hydrogen concentrations is disproportionaly strong. The reason for such a behaviour is the reversible hydrogen decomposition reaction catalysed by palladium, $H_{2}$ ↔ $2H$. The position of equilibrium is defined by the chemical equilibrium constant $K = C\left( H \right)^{2} /C\left( {H_{2} } \right)$, i.e., $C\left( H \right)\sim \sqrt {C\left( {H_{2} } \right)}$. Since the sensor response is determined by the concentration of hydrogen atoms, the following relation is predicted: $a_{PC1} \sim \sqrt {C\left( {H_{2} } \right)}$. This root dependence, known as Sievert’s law^42^, was observed in our experiments. To show this, we extracted the experimental spectra corresponding to stationary level of $a_{PC1}$ at each concentration (Fig. 4b) and averaged the sensor response found with PCA. Such $\left\langle {a_{PC1} } \right\rangle$ vs. $C\left( {H_{2} } \right)$ dependence appeared a straight line on the square root scale for $C\left( {H_{2} } \right)$ (Fig. 4c), confirming the predicted root dependence and meaning the sensor calibration curve. A sensor resolution defined as a standard error corresponding to the deviation of the known hydrogen concentration from the calibration curve was evaluated to be of 40 ppm. This value is illustrated by an error hystogram (Fig. 4d). The ‘root sensitivity’ makes the sensor successfully working with both low and high hydrogen concentrations, thereby increasing its working range.

**Figure 5:**
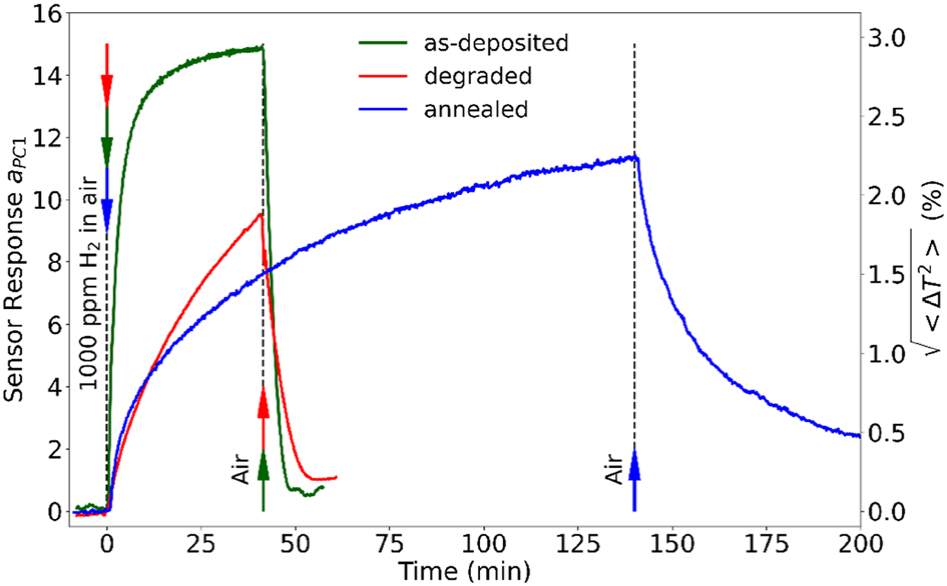
Time-dependent response of the as-deposited (green curve), degraded sensor (red curve) and annealed sensor (blue curve) when exposed to 1000 ppm of hydrogen in air.

Stable response of a sensing element over its lifetime is a vital parameter of any sensor and is one of the major requirements to the sensor operation. In order to study the sensor stability, we compared responses to 1000 ppm of hydrogen in air just after the deposition and after 4 months of intensive use (Fig. 5, green and red curves). One can see that functionality of the degraded sensor has remained, although the response has become slower and weaker. The response time was determined by fitting the time dependence of the response, $a_{PC1} \left( t \right)$, with the sum of two exponential functions: $a_{PC1} \left( t \right) = a_{1} \exp \left( { - t/\tau_{1} } \right) + a_{2} \exp \left( { - t/\tau_{2} } \right)$. In all our approximations, the second term was 5–10 times slower than the first one, therefore, we consider the first term as characterizing the sensor response time (Table 1). The degradation has led to 2 times increase in the $\tau_{1}$ response time.

**Table 1:** Response time of the sensor to 1000 ppm of hydrogen in air.

Sensors	Response time $\tau_{1}$ (min)
As-deposited	$2.10 \pm 0.06$
Degraded	$4.42 \pm 0.18$
Annealed	$3.63 \pm 0.08$

The degradation may be associated with poisoning of the catalyst, deterioration of the WO_3_/Pd interface, or degradation of WO_3_ gasochromic properties. As for the first assumption, palladium operation could be recovered by annealing as reported in Ref.^43^. This is why in our experiments, the sensor was annealed at 200 °C for 1 h. However, we did not observe any substantial improvements (Fig. 5, blue line), except for a slight decrease in the response time. Presumably, a difference from Ref.^43^ was the use of the ultrathin catalyst film in our nanostructure. In our opinion, the most likely mechanism restricting the sensor functionality was degradation of the ultrathin palladium film adhesion due to multiple cycles of adsorption/desorption of hydrogen with the change of the palladium volume. This means that the most likely way to prolong the lifetime of the sensing element is using an adhesive sublayer under the catalyst or replacing pure palladium by its alloy^44–47^. One can also deposit a polymer coating above the palladium film, since the latter presumably has an island structure and will be “glued” to substrate. However, the polymer should be permeable to hydrogen. Another possible approach is a pretreatment of the ${\text{WO}}_{3}$ surface before the palladium deposition (e.g., by plasma).

## Humidity and temperature effects on the sensor selectivity

Metal oxide-based gas sensors are known to be sensitive to humidity and temperature^48–50^. Therefore, a practical hydrogen sensor must differentiate its selective response that can be masked by other factors^51–54^. In this section, we show that the use of two principal components allows achieving the selectivity to hydrogen exposure.

**Figure 6:**
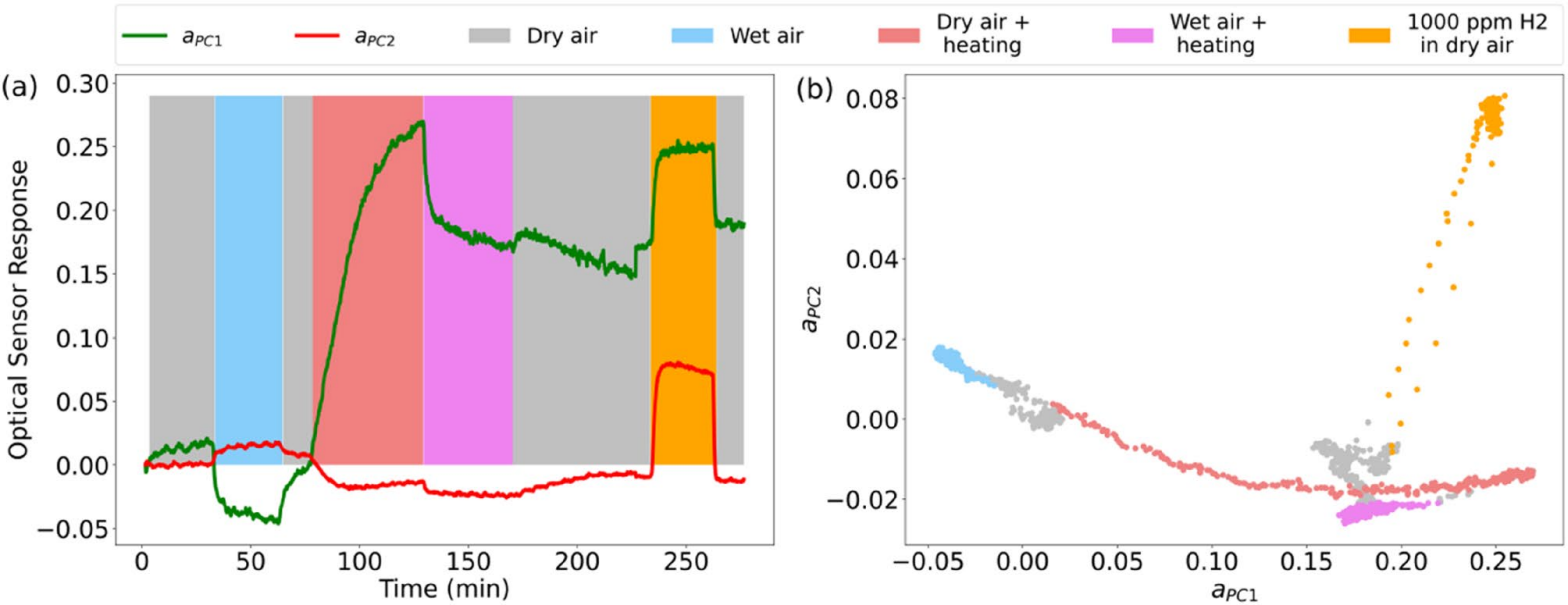
(**a**) Response of the as-deposited sensor in terms of the first ($a_{PC1}$, green) and second. ($a_{PC2}$, red) principal components measured at different conditions and (**b**) a corresponding diagram of $a_{PC1}$ vs. $a_{PC2}$. Responses to wet air (blue) with 30% RH, to heating up to 60 °C (pink), to both humidity and heating simultaneously (magenta), and to 1000 ppm of hydrogen in dry air at room temperature (orange) are shown.

In additional experiments, we have studied an influence of humidity and temperature on the sensor response and processed these results by PCA. As-deposited sensor sample was subsequently subjected to the following conditions: (i) dry air of the room temperature, (ii) wet air of a relative humidity (RH) of 30%, (iii) heating up to 60 °C, (iv) both humidity and heating simultaneously, and (v) 1000 ppm of hydrogen in dry air at room temperature (bars in Fig. 6a). By processing the obtained spectra, principal components were built (Fig. S5 in Supplementary material). Let us note that the principal components differ from those obtained in the previous section since they are built based on another experimental dataset.

The humidity and temperature (blue and red regions in Fig. 6a, respectively) caused changes of the first principal component, $a_{PC1}$, of different signs. The same was for the amplitude of the second principal component, $a_{PC2}$, although its response was much weaker. At changing both humidity and temperature, $a_{PC1}$ expectedly showed a differential response (pink region in Fig. 6a), whereas $a_{PC2}$ did not demonstrate any clear behavior due to its weak response. Therefore, the amplitude of $a_{PC1}$ was affected by each of steps i–v.

Interestingly, in the presence of hydrogen (orange region in Fig. 6a) $a_{PC2}$ showed a strong response. The same can be seen for $a_{PC1}$, although this can be confused with the effect of heating. As for $a_{PC2}$, it demonstrated better selectivity to hydrogen than $a_{PC1}$. However, this selectivity is not fully certain, and the best choice is a simultaneous analysis of the both components. Figure 6b illustrates an observation of a mutual change of the principal components to different impacts as a diagram of $a_{PC1}$ vs. $a_{PC2}$. On this diagram, the response to hydrogen exposure (orange dots) is markedly differed by its trend.

Why does the second principal component change significantly to hydrogen only, whereas all the impacts change the first principal component? Such behavior is observed because both humidity and temperature lead to significant spectral shifts of the resonance, whereas the changes in the resonance intensity are small. On the contrary, the second principal component (Fig. S5 in Supplementary material) describes a change of the resonance intensity—the main transformation in spectra caused by hydrogen.

## Conclusions

In this manuscript, a hydrogen sensor based on a 1D nanostructure comprising a dielectric grating (Al_2_O_3_), gasochromic oxide (WO_3_) and catalyst (Pd) is proposed. The Pd catalyst film was as thin as 1 nm that allowed to significantly decrease absorption in the nanostructure. As a result, a high-Q guided-mode resonance was observed at normal incidence. Transmission spectra were measured at 0–0.12% of hydrogen in air or in nitrogen. It was shown that, the sensitivity to hydrogen was 7 times lower in air than in nitrogen atmosphere. The detection limit of 100 ppm of H_2_ was demonstrated.

Sensor response was treated by the principal component analysis dealing with an integral spectral response and, thereby, carrying out noise averaging. A growth of sensitivity at small hydrogen concentrations was observed. It is shown that the reason for such a behavior is the hydrogen decomposition reaction catalyzed by palladium, which follows the root dependence known as Sievert’s law.

Selectivity to hydrogen was proved by PCA when changing humidity of the exposing atmosphere and heating the sensor.

Long term stability of the sensor was investigated. Although the changes in the response magnitude and time were observed after 4 months and dozens of the redox circles, the sensor retained its functionality.

## Supplementary Information

Supplementary Information.

## Data Availability

The datasets used and/or analyzed during the current study available from the corresponding author on reasonable request.

## References

[1] Hosseini SE, Wahid MA (2016). Hydrogen production from renewable and sustainable energy resources: Promising green energy carrier for clean development. Renew. Sustain. Energy Rev..

[2] Seiyama T, Kato A, Fujiishi K, Nagatani M (1962). A new detector for gaseous components using semiconductive thin films. Anal. Chem..

[3] Miller DR, Akbar SA, Morris PA (2014). Nanoscale metal oxide-based heterojunctions for gas sensing: A review. Sens. Actuators B Chem..

[4] Reyes L (2006). Gas sensor response of pure and activated WO3 nanoparticle films made by advanced reactive gas deposition. Sens. Actuators B Chem..

[5] Li Z (2018). Resistive-type hydrogen gas sensor based on TiO2: A review. Int. J. Hydrogen Energy.

[6] Hodgkinson J, Tatam RP (2012). Optical gas sensing: A review. Meas. Sci. Technol..

[7] Mirzaei A, Kim J-H, Kim HW, Kim SS (2019). Gasochromic WO3 nanostructures for the detection of hydrogen gas: An overview. Appl. Sci..

[8] Tittl A, Giessen H, Liu N (2014). Plasmonic gas and chemical sensing. Nanophotonics.

[9] Granqvist CG (1995). Handbook of Inorganic Electrochromic Materials.

[10] Ndaya CC, Javahiraly N, Brioude A (2019). Recent advances in palladium nanoparticles-based hydrogen sensors for leak detection. Sensors.

[11] Ito K, Ohgami T (1992). Hydrogen detection based on coloration of anodic tungsten oxide film. Appl. Phys. Lett..

[12] Sekimoto S (2000). A fiber-optic evanescent-wave hydrogen gas sensor using palladium-supported tungsten oxide. Sens. Actuators B Chem..

[13] Benson, D. K. *et al.* In *Advanced Sensors and Monitors for Process Industries and the Environment.* 185–202 (International Society for Optics and Photonics).

[14] Watanabe T, Okazaki S, Nakagawa H, Murata K, Fukuda K (2010). A fiber-optic hydrogen gas sensor with low propagation loss. Sens. Actuators B Chem..

[15] Tournier G, Pijolat C (1999). Influence of oxygen concentration in the carrier gas on the response of tin dioxide sensor under hydrogen and methane. Sens. Actuators B Chem..

[16] Yamaguchi Y, Imamura S, Ito S, Nishio K, Fujimoto K (2015). Influence of oxygen gas concentration on hydrogen sensing of Pt/WO3 thin film prepared by sol–gel process. Sens. Actuators B Chem..

[17] Hyodo T, Yamashita T, Shimizu Y (2015). Effects of surface modification of noble-metal sensing electrodes with Au on the hydrogen-sensing properties of diode-type gas sensors employing an anodized titania film. Sens. Actuators B Chem..

[18] Li D, Wu G, Gao G, Shen J, Huang F (2011). Ultrafast coloring-bleaching performance of nanoporous WO_3_–SiO_2_ gasochromic films doped with Pd catalyst. ACS Appl. Mater. Interfaces.

[19] Yamaguchi Y, Nemoto C, Ito S, Nishio K, Fujimoto K (2015). Improvement of hydrogen gas sensing property of the sol–gel derived Pt/WO3 thin film by Ti-doping. J. Ceram. Soc. Jpn..

[20] Lin Y-M, Gao J-J, Chen K-P, Huang C-H, Huang C-S (2020). A novel hydrogen sensor based on a guided-mode resonance filter. IEEE Sens. J..

[21] Sahoo PK, Sarkar S, Joseph J (2017). High sensitivity guided-mode-resonance optical sensor employing phase detection. Sci. Rep..

[22] Triggs GJ (2017). Chirped guided-mode resonance biosensor. Optica.

[23] Foland S, Swedlove B, Nguyen H, Lee J-B (2012). One-dimensional nanograting-based guided-mode resonance pressure sensor. J. Microelectromech. Syst..

[24] Merzlikin AM, Kuznetsov EV, Baryshev AV (2018). Magneto-optical device based on polarization sensitivity for perspective biosensing applications. IEEE Sens. J..

[25] Baryshev A, Merzlikin A (2014). Approach to visualization of and optical sensing by Bloch surface waves in noble or base metal-based plasmonic photonic crystal slabs. Appl. Opt..

[26] Kornienko VV (2020). Machine learning for optical gas sensing: A leaky-mode humidity sensor as example. IEEE Sens. J..

[27] Saetchnikov, A. V., Tcherniavskaia, E. A., Saetchnikov, V. A. & Ostendorf, A. Deep-learning powered whispering gallery mode sensor based on multiplexed imaging at fixed frequency (2020).

[28] Chubchev ED, Tomyshev KA, Nechepurenko IA, Dorofeenko AV, Butov OV (2022). Machine learning approach to data processing of TFBG-assisted SPR sensors. J. Lightw. Technol..

[29] Huang Z (2022). Improving accuracy and sensitivity of a tilted fiber bragg grating refractometer using cladding mode envelope derivative. J. Lightw. Technol..

[30] Ghosh R, Baker MB, Lopez R (2010). Optical properties and aging of gasochromic WO_3_. Thin Solid Films.

[31] Özkan E, Tepehan F (2001). Optical and structural characteristics of sol–gel-deposited tungsten oxide and vanadium-doped tungsten oxide films. Sol. Energy Mater. Sol. Cells.

[32] Saygin-Hinczewski D, Hinczewski M, Sorar I, Tepehan FZ, Tepehan GG (2008). Modeling the optical properties of WO3 and WO_3_–SiO_2_ thin films. Sol. Energy Mater. Sol. Cells.

[33] Von Rottkay K, Rubin M, Wen S-J (1997). Optical indices of electrochromic tungsten oxide. Thin Solid Films.

[34] Nechepurenko IA (2021). Evaluating the response time of an optical gas sensor based on gasochromic nanostructures. Sensors.

[35] Kulikova DP (2020). Optical properties of tungsten trioxide, palladium, and platinum thin films for functional nanostructures engineering. Opt. Express.

[36] Rodionov, I. A. *et al.* Mass production compatible fabrication techniques of single-crystalline silver metamaterials and plasmonics devices. In *Proceedings SPIE 10343. Metamaterials, Metadevices, and Metasystems 2017*. 1034337 (2017).

[37] Rodionov IA (2019). Quantum engineering of atomically smooth single-crystalline silver films. Sci. Rep..

[38] Boginskaya I (2019). SERS-Active substrates nanoengineering based on e-beam evaporated self-assembled silver films. Appl. Sci..

[39] Dobronosova AA (2019). Low-damage reactive ion etching of nanoplasmonic waveguides with ultrathin noble metal films. Appl. Sci..

[40] Wang S, Magnusson R (1993). Theory and applications of guided-mode resonance filters. Appl. Opt..

[41] Szeghalmi A, Kley EB, Knez M (2010). Theoretical and experimental analysis of the sensitivity of guided mode resonance sensors. J. Phys. Chem. C.

[42] Dornheim M, Moreno-Pirajan JC (2011). Thermodynamics—Interaction Studies—Solids, Liquids and Gases.

[43] Luo JY (2012). Study of the catalyst poisoning and reactivation of Pt nanoparticles on the surface of WO_3_ nanowire in gasochromic coloration. Sens. Actuators B Chem..

[44] Chen K, Yuan D, Zhao Y (2021). Review of optical hydrogen sensors based on metal hydrides: Recent developments and challenges. Opt. Laser Technol..

[45] Bannenberg L, Schreuders H, Dam B (2021). Tantalum-palladium: Hysteresis-free optical hydrogen sensor over 7 orders of magnitude in pressure with sub-second response. Adv. Funct. Mater..

[46] Darmadi I, Khairunnisa SZ, Tomecek D, Langhammer C (2021). Optimization of the composition of PdAuCu ternary alloy nanoparticles for plasmonic hydrogen sensing. ACS Appl. Nano Mater..

[47] Nishijima Y (2017). Optical readout of hydrogen storage in films of Au and Pd. Opt. Express.

[48] Steele JJ, Taschuk MT, Brett MJ (2008). Nanostructured metal oxide thin films for humidity sensors. IEEE Sens. J..

[49] Yamazoe N, Shimizu Y (1986). Humidity sensors: Principles and applications. Sens. Actuators.

[50] Wang C, Yin L, Zhang L, Xiang D, Gao R (2010). Metal oxide gas sensors: Sensitivity and influencing factors. Sensors.

[51] Hossein-Babaei F, Ghafarinia V (2010). Compensation for the drift-like terms caused by environmental fluctuations in the responses of chemoresistive gas sensors. Sens. Actuators B Chem..

[52] Ziyatdinov A (2010). Drift compensation of gas sensor array data by common principal component analysis. Sens. Actuators B Chem..

[53] Vergara A (2012). Chemical gas sensor drift compensation using classifier ensembles. Sens. Actuators B Chem..

[54] Padilla M (2010). Drift compensation of gas sensor array data by orthogonal signal correction. Chemom. Intell. Lab. Syst..

