# Supplementary material for: Optical hydrogen sensing with high-Q guided-mode resonance of Al2O3/WO3/Pd nanostructure
Source: Sci Rep. 2023 Jan 17;13:890. doi: 10.1038/s41598-023-28204-z (PMC9845354; doi:10.1038/s41598-023-28204-z)
Supplement: Supplementary file 1 — Supplementary Information. [file 41598_2023_28204_MOESM1_ESM.docx]

**Supplementary material for “Optical hydrogen sensing with high-Q guided-mode resonance of Al_2_O_3_/WO_3_/Pd nanostructure Principal Component Analysis Applied to Spectra”**

D.P. Kulikova^1,2^, Y.M. Sgibnev^1^, G.M. Yankovskii^1^, E.D. Chubchev^1^, E. S. Lotkov^1,3^, D.A. Ezenkova^1,3^, A.A. Dobronosova^1,3^, A.S. Baburin^1,3^, I.A. Rodionov^1,3^, I.A. Nechepurenko^1,4,5^, A.V. Baryshev^1^, A.V. Dorofeenko^1,5,6^

^1^*Dukhov Research Institute of Automatics (VNIIA), Moscow, Russia*

^2^*Lomonosov Moscow State University, Faculty of Physics*

^3^*FMN Laboratory, Bauman Moscow State Technical University*

^4^*Kotelnikov Institute of Radioengineering and Electronics RAS, Moscow, Russia*

^5^*Moscow Institute of Physics and Technology, Dolgoprudny, Moscow Region, Russia*

^6^*Institute for Theoretical and Applied Electromagnetics RAS, Moscow Region, Russia*

**1. Details of the full-wave simulation of nanostructure**

The nanostructure was modeled by the finite element method (FEM) with COMSOL Multiphysics software. A single unit cell was simulated with the periodic boundary conditions at the side boundaries. A non-uniform mesh with an upper bound of 20 nm was used (Fig. S1). The minimal mesh element was about 0.3 nm, which was enough to resolve the field in ultrathin palladium film.


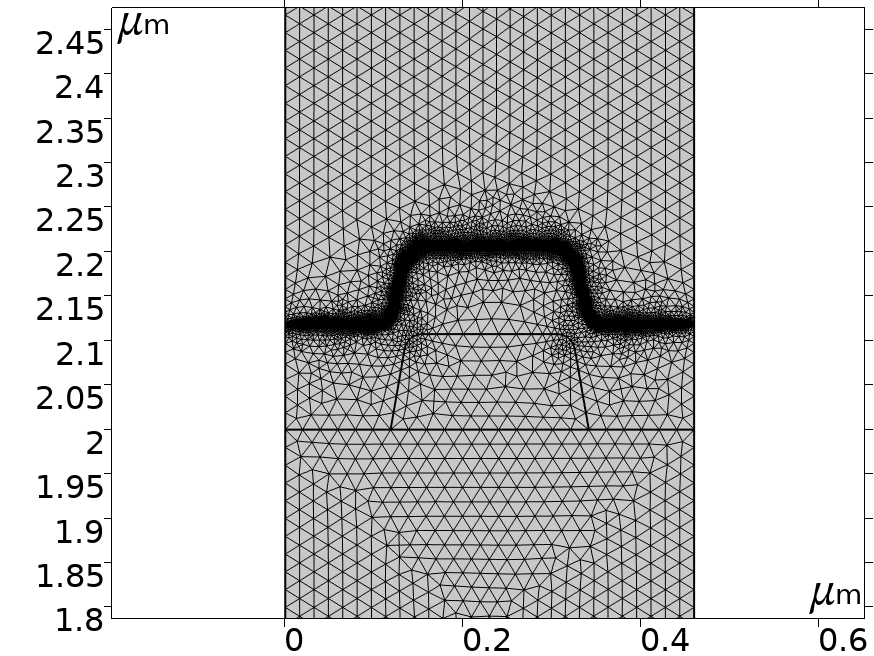


Fig. S1. Discretization mesh of the sensing nanostructure.

The nanostructure was excited by a plane wave at normal incidence, which was generated by an instrument of the periodic port in Comsol. This port was located at the top boundary of the simulation region. Another periodic port without excitation was located at the bottom boundary, which produced a reflectionless condition of the wave from the simulation region. The vertical size of the simulation region was set to several wavelengths in order to avoid interaction of near fields with boundaries. As a result, transmission spectra of the nanostructure were calculated.

**2. Elemental analysis of the nanostructure**

We have carried out an elemental analysis of the nanostructure by energy dispersive spectroscopy (EDS). We show two pictures: (*i*) the one without oxygen (Fig. S2a), where one observes Si in the quartz substrate, Al in the Al_2_O_3_ grating, W in WO_3_ film and Pd atop the nanostructure; (*ii*) the one with oxygen, which appears in all the oxides (Fig. S2b).


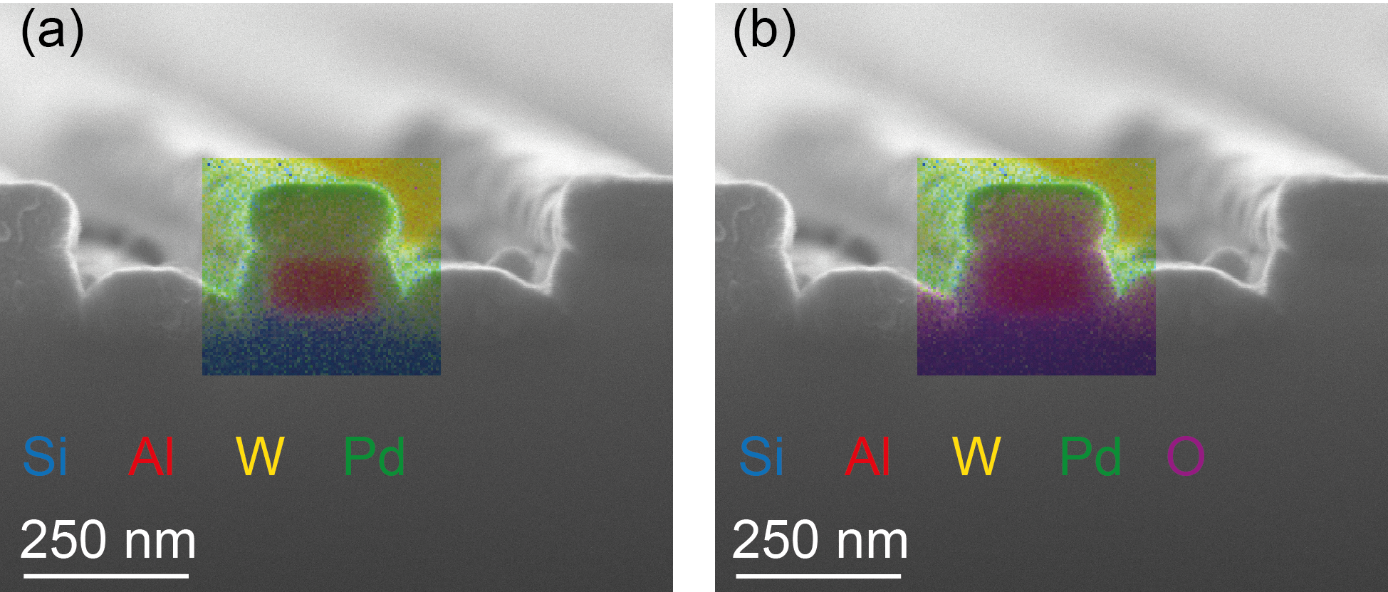


Fig. S2. EDS maps without (a) and with (b) oxygen shown.

It is necessary to note that the nanostructure shown here differs from the one shown in Fig. 1 of the main paper (the latter sample was used in obtaining all the results). Both samples were fabricated with the same geometrical parameters, and their difference was due to technological discrepancy.

**3. Processing the sensor data with principal component analysis (PCA)**

To gain full information from the sensor response, principal component analysis^1^ was applied to a wavelength range from $\lambda_{1}=667$ nm to $\lambda_{2}=687$ nm (Fig. S3), in which transmissivity was largely changed versus a change in hydrogen concentration due to the guided mode resonance. In the framework of principal component analysis (PCA), each transmission spectrum is approximated by a linear combination of principal components,

$T\left(\lambda,C\right)\approx T\left(\lambda,C=0\right)+\sum_{i}^{q} a_{PCi}\left(C\right)PC_{i}\left(\lambda\right)$,

where $C=C(H_{2})$ denotes the hydrogen concentration, $a_{PCi}\left(C\right)$ is the decomposition coefficient, $q$ is the number of principal components used in the approximation.


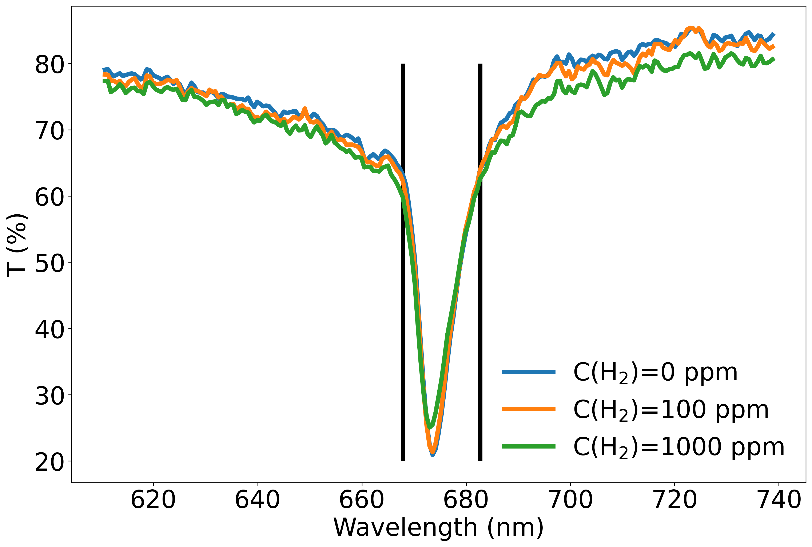


Fig. S3. Transmission spectra of the sample taken for the E_⊥_-polarized light at different hydrogen concentrations. Black vertical lines denote limits of the wavelength range used for PCA.

The approximation error is minimal under a condition that $PC_{i}$ are the eigenvectors of the covariance matrix in total of $q$ largest eigenvalues $\sigma$. Since principal components are the eigenvectors of the symmetrical matrix, they are mutually orthogonal, and the decomposition coefficients $a_{PCi}\left(C(H_{2})\right)$ can be found as

$$\begin{matrix} a_{PC1}\left(C\right)=\left(PC_{1}\cdot\left[T\left(C\right)-T\left(C=0\right)\right]\right) \\ =\sum_{k=1}^{N} PC_{1}\left(\lambda_{k}\right)\left[T\left(\lambda_{k},C\right)-T\left(\lambda_{k},C=0\right)\right]. \end{matrix}$$

Here, the normalization condition $\left\|PC_{1}\right\|^{2}=\sum_{k=1}^{N} PC_{1}^{2}\left(\lambda_{k}\right)=1$ is assumed. We had *N = 26* experimental points in a spectral range from 667 to 687 nm.

To calculate the principal components, we used a set of 1542 spectra obtained in the experiment, in which the hydrogen concentration of 100 ppm and 1000 ppm was applied to the sensor (Fig. 4a of the main text). In Fig. S4a, magnitudes of the eigenvalues of the covariance matrix are shown. Since the first eigenvalue was significantly larger than all the other eigenvalues, the principal component corresponding to the largest eigenvalue was enough to account for a good approximation of the experimental spectra. The plot of $PC_{1}$ is shown in Fig. S4b.

To make $a_{PC1}\left(C\right)$ physically sound, we find a relation of this quantity to an average change in transmissivity in the $\lambda_{1}\leq\lambda\leq\lambda_{2}$ spectral range:

$$\begin{matrix} \left\langle\Delta T^{2}\right\rangle=\frac{\Delta\lambda}{\lambda_{2}-\lambda_{1}}\sum_{k=1}^{N} \left(T\left(\lambda_{k},C\right)-T\left(\lambda_{k},C=0\right)\right)^{2}= \\ =\frac{\Delta\lambda}{N\Delta\lambda}\sum_{k=1}^{N} \left(T\left(\lambda_{k},C\right)-T\left(\lambda_{k},C=0\right)\right)^{2}=\frac{1}{N}\left\|T\left(C\right)-T\left(C=0\right)\right\|^{2}. \end{matrix}$$

Substituting Eq. (S1) into Eq. we find

$$\left\langle\Delta T^{2}\right\rangle=\frac{1}{N}\left\|\sum_{i}^{q} a_{PCi}\left(C\right)PC_{i}\left(\lambda\right)\right\|^{2}$$

and, leaving the first principal component only, $\left\langle\Delta T^{2}\right\rangle\approx\frac{1}{N}a_{PC1}\left(C\right)^{2}\left\|PC_{1}\left(\lambda\right)\right\|^{2}$, we obtain the following relation:

$\sqrt{\left\langle\Delta T^{2}\right\rangle}\approx\left|a_{PC1}\left(C\right)\right|/\sqrt{N}$.

It is worth noting that equality can be nearly achieved in Eq. if the eigenvalue corresponding to $PC_{1}$ is much more larger in magnitude than all other eigenvalues.


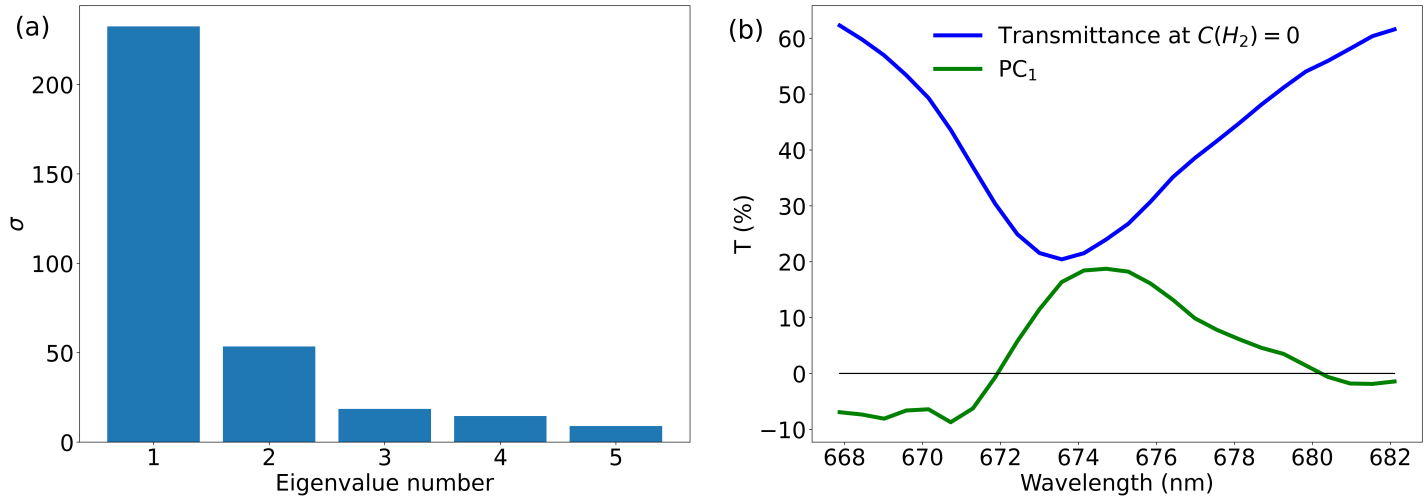


Fig. S4. (a) Magnitudes of eigenvalues of the covariance matrix. (b) The transmission spectrum $T\left(\lambda\right)$, obtained in the experiment in air (blue curve), and $PC_{1}\left(\lambda\right)$ (green curve).

In Sec. 5, PCA was applied to a different dataset obtained when sensor was subjected to dry air of the room temperature, to wet air, to heating up to 60  C, to both humidity and heating simultaneously, and after that to 1000 ppm of hydrogen. In Fig. S5a, the eigenvalues of covariance matrix computed for this dataset are shown. The first two eigenvalues are the largest and, therefore, the associated principal components should be taken into account.


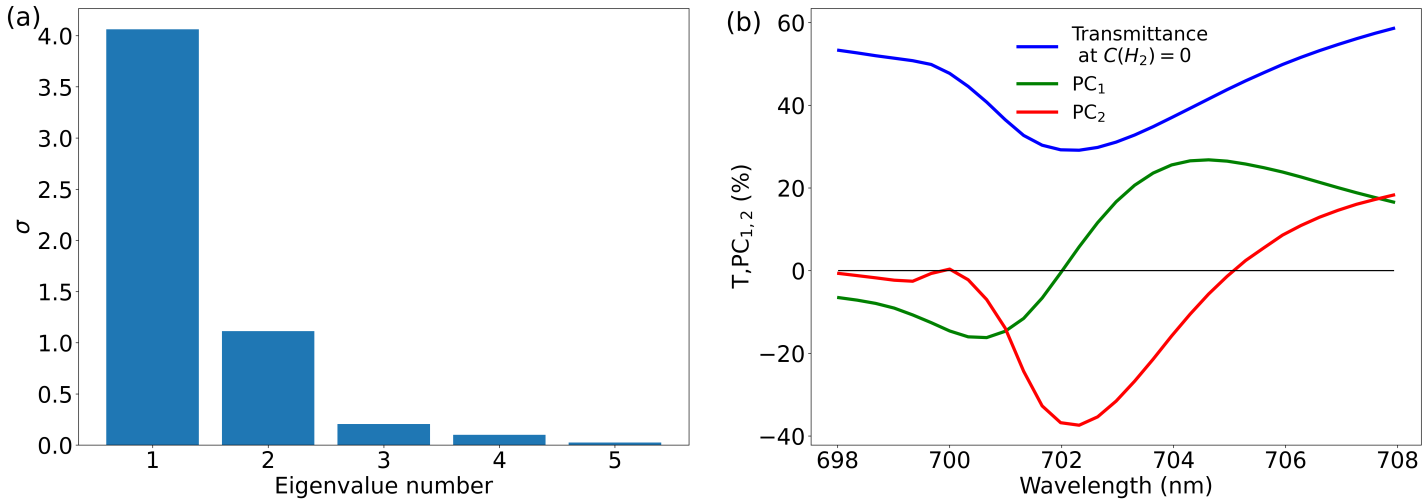


Fig. S5. (a) Magnitudes of eigenvalues of the covariance matrix. (b) The transmission spectrum $T\left(\lambda\right)$, obtained in the experiment in air (blue curve), $PC_{1}\left(\lambda\right)$ (green curve), and $PC_{2}\left(\lambda\right)$ (red curve)

The principal components $PC_{1}\left(\lambda\right)$ and $PC_{2}\left(\lambda\right)$ are shown in Fig. S5b. The first principal component $PC_{1}\left(\lambda\right)$ is similar to the wavelength derivative of the first spectrum $\partial T\left(\lambda,C\left(H_{2}\right)=0\right)/\partial\lambda$ and, therefore, the change in $a_{PC1}$ leads to the shift of the resonant dip. The second principal component has a shape similar to the transmission spectrum itself, so that the change in $a_{PC2}$ corresponds to a change of the depth of the resonance.

As shown in the paper, the shift of the resonant wavelength can be caused by humidity, heating, or by the presence of hydrogen, however, the presence of hydrogen also leads to the change of the depth in the transmittance resonance. Therefore, the sensor response to hydrogen can be separated from other impacts.

^1^ A. N. Gorban, B. Kégl, D. C. Wunsch, and A. Y. Zinovyev, *Principal manifolds for data visualization and dimension reduction*. (Springer, 2008).
